# Transmission and lineage displacement drive rapid population genomic flux in cystic fibrosis airway infections of a *Pseudomonas aeruginosa* epidemic strain

**DOI:** 10.1099/mgen.0.000167

**Published:** 2018-03-16

**Authors:** David Williams, Joanne L. Fothergill, Benjamin Evans, Jessica Caples, Sam Haldenby, Martin J. Walshaw, Michael A. Brockhurst, Craig Winstanley, Steve Paterson

**Affiliations:** ^1^​ Institute of Integrative Biology, University of Liverpool, Liverpool, UK; ^2^​ Institute of Infection and Global Health, University of Liverpool, Liverpool, UK; ^3^​ Liverpool Heart and Chest Hospital, NHS Foundation Trust, Liverpool, UK; ^4^​ Animal and Plant Sciences, University of Sheffield, Sheffield, UK

**Keywords:** pathogen evolution, chronic infection, cystic fibrosis, *Pseudomonas*, competition

## Abstract

*Pseudomonas aeruginosa* chronic infections of cystic fibrosis (CF) airways are a paradigm for within-host evolution with abundant evidence for rapid evolutionary adaptation and diversification. Recently emerged transmissible strains have spread globally, with the Liverpool Epidemic Strain (LES) the most common strain infecting the UK CF population. Previously we have shown that highly divergent lineages of LES can be found within a single infection, consistent with super-infection among a cross-sectional cohort of patients. However, despite its clinical importance, little is known about the impact of transmission on the genetic structure of these infections over time. To characterize this, we longitudinally sampled a meta-population of 15 genetic lineages within the LES over 13 months among seven chronically infected CF patients by genome sequencing. Comparative genome analyses of *P. aeruginosa* populations revealed that the presence of coexisting lineages contributed more to genetic diversity within an infection than diversification *in situ*. We observed rapid and substantial shifts in the relative abundance of lineages and replacement of dominant lineages, likely to represent super-infection by repeated transmissions. Lineage dynamics within patients led to rapid changes in the frequencies of mutations across suites of linked loci carried by each lineage. Many loci were associated with important infection phenotypes such as antibiotic resistance, mucoidy and quorum sensing, and were repeatedly mutated in different lineages. These findings suggest that transmission leads to rapid shifts in the genetic structure of CF infections, including in clinically important phenotypes such as antimicrobial resistance, and is likely to impede accurate diagnosis and treatment.

## Data Summary

1. DNA sequence data are available from the European Nucleotide Archive under accessions PRJEB6642 and PRJEB20763.

**Impact Statement**

Cystic fibrosis (CF) patients are susceptible to lifelong infections of the airways caused by *Pseudomonas aeruginosa*. In the UK there is a widespread CF transmissible strain [the Liverpool Epidemic Strain (LES)], which is associated with poorer patient health. Genetic diversity within the lung makes CF infections harder to treat but how transmission between patients affects diversity is unknown. Genome sequencing of *P. aeruginosa* populations from a cohort of LES-infected CF patients over one year showed that most genetic diversity within infections was due to the acquisition of genetically diverged *P. aeruginosa* lineages by transmission. The frequency and identity of these lineages within infections changed rapidly, causing wholesale shifts in a suite of genes linked to pathology. Transmission increases genetic diversity in CF infections and boosts the rate and magnitude of evolutionary change, posing challenges for treatment.

## Introduction

*Pseudomonas aeruginosa* has typically been considered an opportunistic pathogen that lives in the environment but can also infect susceptible individuals [1]. In particular, patients with cystic fibrosis (CF) are susceptible to chronic bacterial infections of the airway, and such infections, once established, are extremely difficult to clear, can last many years and, despite antibiotic and other interventions, are associated with continued decline of lung function leading to death or lung transplant [2]. Chronic infections of CF airways by *P. aeruginosa* strains provide a powerful model to understand the evolutionary processes associated with the adaptation of bacterial pathogens to survive within the human host [3, 4]. DNA sequencing of longitudinal samples of environmentally acquired infections has revealed a number of genetic changes associated with adaptation, including switch to a mucoid phenotype, loss of quorum sensing and motility, and development of auxotrophy, hypermutability and antibiotic resistance [5, 6]. It is now apparent, however, that, alongside environmentally acquired strains, patient-to-patient transmission is also common, predominantly through a subset of specialized pathogen strains – termed transmissible or epidemic strains – that appear to have adapted to both survive within the human airway and retain the ability to transmit between patients [7–9]. Transmissibility (i.e. the ability to transmit between patients) appears to have evolved repeatedly and independently, since genetically distinct transmissible strains have been reported from studies in Australia, Belgium, Canada, Denmark and the UK [9–11].

The Liverpool Epidemic Strain (LES) is the most common transmissible strain in the UK, has also been found in North America, and is associated with both higher virulence in laboratory models and poorer patient outcomes than environmentally acquired strains [12–15]. Little is known about how transmission affects the population genetic structure of CF infections. We have previously demonstrated that the population of LES bacteria infecting a single patient is composed of a diversity of genetically and phenotypically distinct clones, which differ in traits believed to be associated with adaptation to human airways [6, 14, 16–18]. Whole genome sequencing of multiple clones recovered from a single sample shows that diversification, due to *de novo* mutation, is a prominent feature of genome evolution in chronic infections for the LES [18]. Using a cross-sectional sample of multiple patients attending the same clinic, we have also demonstrated coexistence of multiple, divergent lineages of the LES that are inconsistent with a single source of infection. Thus, patients can be infected with multiple lineages of the LES, either as part of the original inoculum or as a result of distinct transmission events (super-infection). Unlike in environmentally acquired infections, for transmissible strains genetic diversity within a chronic infection arises both by *in situ* diversification of an infecting clone into a clonal lineage and by coexistence within an infection of multiple clonal lineages acquired by recurrent transmission [18]. There is substantial phenotypic variation between samples of the LES taken from patients at successive time points [16, 17], which suggests that recurrent transmissions may be driving rapid population genomic flux in LES infections of the CF airway.

From a clinical perspective, diversity within an infection makes *P. aeruginosa*, and other pathogens, difficult to treat because an infection may maintain a reservoir of genetic diversity able to evolve rapidly in response to either host immune responses or clinical interventions, including antibiotics [3, 6, 19]. Changes in the genetic structure of the infection through time may also have significant implications for the health of a patient, should the frequency of traits associated with pathology change through time. Unlike *in situ* diversification by *de novo* mutation [4], acquisition of new diversity via transmission is likely to represent a larger-scale alteration to population genetic structure, affecting many loci simultaneously and much more rapidly than would occur via mutation-selection [20], yet little is known about the temporal dynamics of genetic diversity in CF infections caused by transmissible strains. Here we build on our previous work, particularly our genomic analysis of single sputum samples from a group of LES-infected patients [18], to use whole genome sequencing to analyse the genetic structure of chronic *P. aeruginosa* LES infections through a series of longitudinal samples from each of seven patients from the same CF clinic.

## Methods

### Patients and samples

Longitudinal samples were collected from seven adult CF patients, each chronically infected with the *P. aeruginosa* LES, as described previously [16]. Briefly, a sputum sample was collected from each patient at a routine visit to the Regional Adult Cystic Fibrosis Unit in Liverpool, UK, during January 2009. Sputum was treated with an equal volume of Sputasol (Oxoid), incubated at room temperature with shaking at 200 r.p.m. for 15 min, and then cultured on *Pseudomonas* selective agar under aerobic conditions with CN supplement (Oxoid) and 40 LES colonies were selected to maximize colony morphology diversity and identified as described previously [16–18]. Details concerning age, sex and clinical status of patients are summarized in Tables S1 and S2 (available in the online version of this article) and in a previous publication [17]. CF01, CF03, CF05 and CF07 are known to have been LES-positive since at least 1995; CF04 and CF09 since 2004; and CF08 since at least 2008.

### DNA sequencing

DNA was extracted from isolates grown in Luria Broth overnight at 37 °C using the Wizard Genomic DNA Purification Kit (Promega). The purity of the extracted bacterial DNA was assessed using the Nanodrop ND-1000 spectrophotometer and the quantity of bacterial DNA was measured using the Qubit 2.0 Fluorometer following the manufacturer's protocol (Life Technologies). For each sample, two isolates were individually sequenced and equimolar pools of 40 isolates were sequenced (Table S2). An Illumina HiSeq 2000 sequencer was used to generate 100 bp paired reads from the ends of 500 bp fragments. Sequenced read data in fastq files were trimmed for the presence of Illumina adapter sequences using Cutadapt version 1.2.1 [21]. The option -O 3 was used, so the 3′ end of any reads which match the adapter sequence for 3 bp or more is trimmed. The reads are further trimmed using Sickle version 1.2 [22] with a minimum window quality score of 20. Reads shorter than 10 bp after trimming were removed.

### Variant calling

The Genome Analysis Toolkit (GATK) Indel Realigner module was used to realign raw reads around indels [23]. Single nucleotide polymorphism, insertion and deletion discovery was performed with GATK's Unified Genotyper module [23] with sample ploidy *n*=1 or *n*=40 for single and pooled isolates, respectively. GATK was further used to filter sequence variants using standard conservative filtering parameters to provide high-quality variant calls. False positive SNP variants at structural mismatches between sample genome sequence and the reference genome sequence (e.g. an absent prophage sequence) were mitigated by omitting variants at positions where aligned read coverage depth was less than 25 % of the genome-wide median. Contiguous regions greater than 10 bp in the reference genome along which no sequenced reads were aligned were considered missing in the genome from which the reads were derived. SNPs, insertions and deletions reported by GATK within annotated protein coding regions of the reference genome (LESB58) were classified on the basis of potential phenotypic effects using snpEff v3.1 [24].

### Statistical analysis

Genome phylogenies were first reconstructed for single clones using the BioNJ algorithm on raw counts of nucleotide differences between pairs of sequences as implemented in the APE version 3.1–2 for the R statistical computing environment version 3.1.0. In order to validate key bipartitions of the BioNJ-derived phylogeny, phylogenies were also reconstructed by maximum likelihood using the IQ-Tree package [25], ModelFinder for substitution model selection [26] and the UFBoot2 non-parametric bootstrap approximation [27] with 1000 replicates to test clade support. The command used was ‘iqtree-omp -m MFP -st DNA -nt AUTO -bb 1000 –s multiple_sequence_alignment.fna’. Rooting was inferred using LESB58 as an outgroup (shown previously to be suitable; [18]). The k3Pu substitution model [28] modified for unequal base frequencies and with no among-site rate correction was selected based on the Bayesian information criterion. Lineages were defined as coherent sets of isolates with high genetic similarity to each other but divergent from each other based on substitutions on branches within the phylogeny (Supplemental materials and Fig. S1). For each sample, divergent, coexisting lineages were defined as any pair of clades whose respective most recent common ancestors (MRCAs) are further from each other than their shared MRCA is from that of all patient isolates, i.e. of this epidemic. All lineage edges had 100 % bootstrap support by both phylogenetic methods. For pooled samples, SNPs fixed within lineages were assigned on the basis of allele frequencies within the pool and from one or more clone sequences from a sample [18] (Supplemental materials and Fig. S2).

Nucleotide variation within samples – the average number of nucleotide differences between a randomly drawn pair of isolates – due to diversification *in situ* versus fixed differences between lineages was estimated for patient CF3, where five samples containing the same pair of lineages was available. Monte Carlo simulation was used to generate a null distribution for technical variation in lineage proportions due to sampling, which could be contrasted against observed variation between samples.

### Quantitative PCR

Based on the sequencing results, lineage-specific primers were successfully designed to span mutations identified in lineages D and I (Table S3). LESB58 DNA was used as a negative control. To validate the method, 40 individual isolates from sample CF7S1 were subjected to PCR amplification primers for lineage D and 4/40 isolates showed positive amplification, which supports the lineage prediction from the pooled sequencing data.

DNA extracted from sputum samples was used in quantitative PCR assays. Each sputum sample was treated with an equal volume of Sputasol (Oxoid) and incubated at 37 °C for 30 min, with shaking at 200 r.p.m. DNA was prepared from each treated sputum sample (400 µl) using the ‘Qiasymphony Virus/Pathogen DNA extraction kit’ (Qiagen) and the automated QIAsymphony machine (Qiagen; pathogen complex 200 protocol). *P. aeruginosa* total density was quantified using primers specific for *P. aeruginosa* (gyrPA-F1/gyrPA-R1) [29] and specific lineages. All primer sequences and targets are listed in Table S3. Quantitative real-time PCR (qPCR) was performed using the Rotor-Gene Q cycler (Qiagen) and 0.1 ml strip-tubes (Qiagen). Each 25 µl reaction consisted of 1× Rotor Gene SYBR Green PCR Master Mix (Qiagen), specific forward and reverse primers (Table S3), and 2 µl cDNA and molecular-grade water added to a final volume of 25 µl. Thermal cycling was performed as follows: activation of DNA polymerase at 95 °C for 5 min, followed by 40 cycles of 95 °C for 10 s, 60 °C for 15 s and 72 °C for 20 s. Fluorescence data were collected at the end of every 72 °C step. PCR products were subsequently analysed by melting curve analysis ranging from 72 to 95 °C. Each qPCR was performed in triplicate and a standard curve of known copy number was performed in each run. No-template controls were included in each qPCR and contained molecular-grade water instead of template DNA. When necessary, qPCR products were analysed by agarose gel electrophoresis. Obtained data were analysed using the Rotor-Gene Q series software (Qiagen).

## Results

### Genetic structure of *P. aeruginosa* LES lineages

**Fig. 1.**
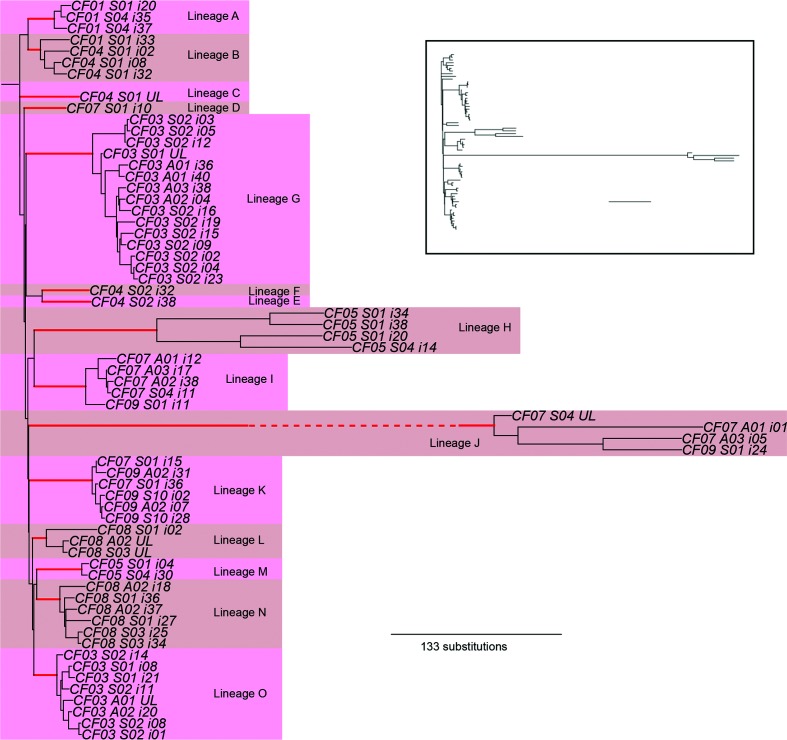
Genetic structure of *Pseudomonas aeruginosa* lineages. The figure shows a rooted neighbour-joining (BioNJ) phylogenetic reconstruction, where labels by each tip give patient, sample and isolate name (i indicates a sequenced isolate and UL an unsampled isolate within a sample inferred from pooled sequencing data; S indicates a sample taken from a patient while clinically stable and A from a patient during an acute exacerbation). Divergent coexisting lineages are defined as exhibiting greater divergence from each other relative to the divergence between their most recent common ancestor (MRCA) and that of the MRCA of all patient isolates. Edges defining lineages are shown in red and have 100 % bootstrap support in all cases. See Fig. S1 for an illustrative example. In each case, lineages are observed to be separated from any other lineage by at least 30 substitutions and in some cases several hundred. Lineage J has undergone hypermutation and its branch length is abbreviated for clarity on the main plot but shown in the insert panel.

We examined the genetic diversity of chronic *P. aeruginosa* LES infections among a set of CF patients from a single clinic, sampled longitudinally over the course of approximately one year. These patients were in contact with each other but separated from other CF patients. Genome sequencing was performed on a combination of single isolates and pools of isolates from each sample. The phylogenetic relationship among single isolates is shown in Fig. 1 and, consistent with a previous report [18], demonstrates the existence of divergent lineages within the LES populations infecting these patients. The majority of samples contained more than one lineage and some lineages (B, K and I) were shared between patients, which is inconsistent with diversity arising from a single infection event by a single clonal genotype. Given the high potential for transmission of the LES amongst patient cohorts, it is likely that patients acquire novel LES lineages by super-infection [13, 30]. In support of this, patients CF07 and CF09 share a lineage (J) that exhibits a hypermutator phenotype [31] that rapidly accumulates genetic substitutions and so allows a high resolution of their genetic relationship. Here samples clearly cluster by lineage rather than by patient, consistent with transmission of lineages between patients (Fig. 1).

We then sequenced pools of 40 isolates and, by combining with single isolate data as described previously [18], were able to infer a total of 15 lineages among 21 samples from seven patients. Coexisting lineages were detected in 18 of 21 samples. Coexistence of highly diverged lineages within populations is therefore a common feature of infection with this transmissible strain and contributes more to genetic diversity within infections than *in situ* mutation. This is demonstrated in patient CF03, where lineages G and O coexisted in the five samples taken from this patient and contributed more to the genetic diversity (average number of nucleotide differences between a pair of randomly drawn isolates) present within infections than diversification *in situ* (an average of 49.8 fixed lineage differences versus 19.8 mutations via diversification *in situ*; paired *t*-test *t*=10.8, d.f.=4, *P*<0.001, Table 1).

**Table 1.** Analysis of genetic variation due to fixed lineage differences and *in situ* mutation in samples from patient CF03

Sample	Date	Total diversity*	Due to lineage component	Due to *in situ* mutation	Estimated abundance of lineage G†
CF03-S1	06/01/2009	68.5	51.8 (76 %)	16.7 (24 %)	63 %
CF03-S2	20/01/2009	76.9	54.0 (70 %)	22.9 (30 %)	42 %
CF03-A1	25/01/2009	60.2	40.4 (67 %)	19.8 (33 %)	77 %
CF03-A2	27/01/2009	69.1	48.2 (70 %)	20.9 (30 %)	68 %
CF03-A3	03/02/2009	73.1	54.4 (74 %)	18.7 (26 %)	59 %

*Nucleotide diversity; the average number of nucleotide differences between two randomly picked genomes.

†CF03 infections were composed of two lineages, G and O.

### Lineage dynamics within *P. aeruginosa* LES infections

**Fig. 2.**
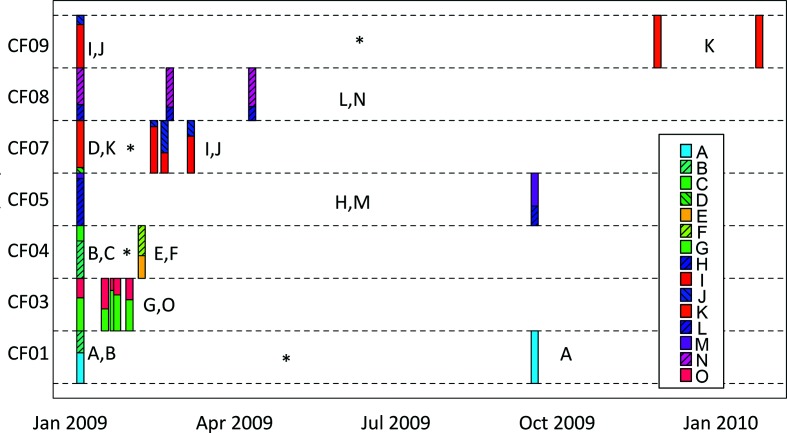
Lineage dynamics within *Pseudomonas aeruginosa* infections. The abundance inferred for each of 15 lineages from pooled sequencing of 40 isolates per sample from longitudinal samples of seven patients is shown. Asterisks indicate periods between samples where lineages are either lost or fall in frequency below the limit of detection from sequencing 40 isolates per sample. Individual lineages are colour-coded as indicated in the insert panel, and their frequencies within sets of 40 isolates per sputum sample indicated with stacked frequency box plots at different sampling time points for each patient (CF01, CF03, CF04, CF05, CF07, CF08, CF09).

Longitudinal sampling revealed rapid fluctuations in the relative frequencies of coexisting lineages within a patient (Fig. 2). For example, samples taken from patient CF03 showed the frequency of lineage O varying between 22 and 58 % over the course of a month, which is significantly greater than expected by sampling variation alone (*P*<0.05 by Monte Carlo simulation). More striking is the loss or replacement of lineages through time. In patient CF07, lineages K and D were observed in the initial January 2009 sample but were not detected in later samples. Thereafter, lineages I and J dominated. Nevertheless, sampling 40 isolates per sample has a limited resolution to detect persistence of lineages at a low abundance. From a binomial distribution, sampling 40 isolates will detect a 0.04 SNP frequency with 80 % power and a 0.07 SNP frequency with 95 % power. To increase resolution further, and to check for errors due to sample mislabelling, we performed qPCR assays to detect lineages I and D from these samples, plus two intermediate, unsequenced sputum samples. Lineage D was confirmed in all samples that were tested by qPCR (Fig. S3), which excludes sample mislabelling as a source of error. Lineage I was present in all samples but was detected only at a low level in January 2009 (compared to total *P. aeruginosa*) whereas levels were substantially higher during the following month (Fig. S4). In contrast, loss of lineage I was observed in patient CF09 (Fig. S4). Here, lineage I was at high frequency in January 2009 but could not be detected by qPCR in sputum samples subsequently and, from sequence data in December 2009 and January 2010, appeared to have been replaced by lineage K. From pooled sequence data (Fig. 2), similar turnover of lineages was observed in patient CF01, where lineage B was lost or fell below detectable levels, and in patient CF04 where lineages B and C fell below detectable levels for lineages E and F to then dominate. Nevertheless, lineage turnover was not ubiquitous, because in three of the seven patients, CF03, CF05 and CF08, the same lineages were detected throughout the study.

### Rapid genetic changes at pathoadaptive loci

**Fig. 3.**
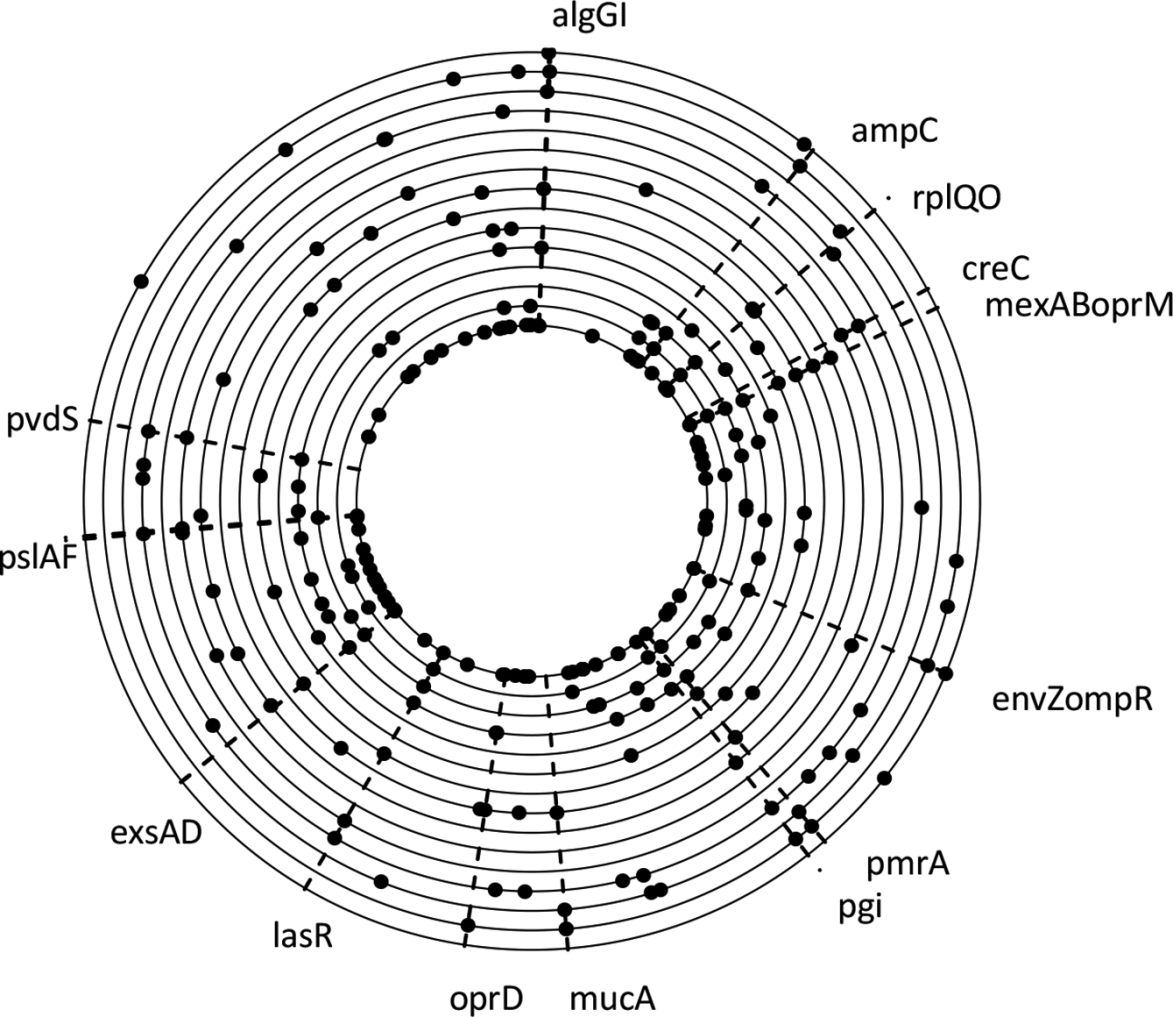
Parallel mutations within loci among *Pseudomonas aeruginosa* lineages. Loci with fixed mutations in two or more lineages are shown. The nucleotide identity of mutations is different in different lineages, indicating independent origins of mutations. All mutations shown result in coding changes. Loci previously implicated in pathoadaptation are highlighted. The 6.6 Mbp genomes of lineages A–O are arranged concentrically, with lineage A innermost, and the origin of replication at the top.

**Fig. 4.**
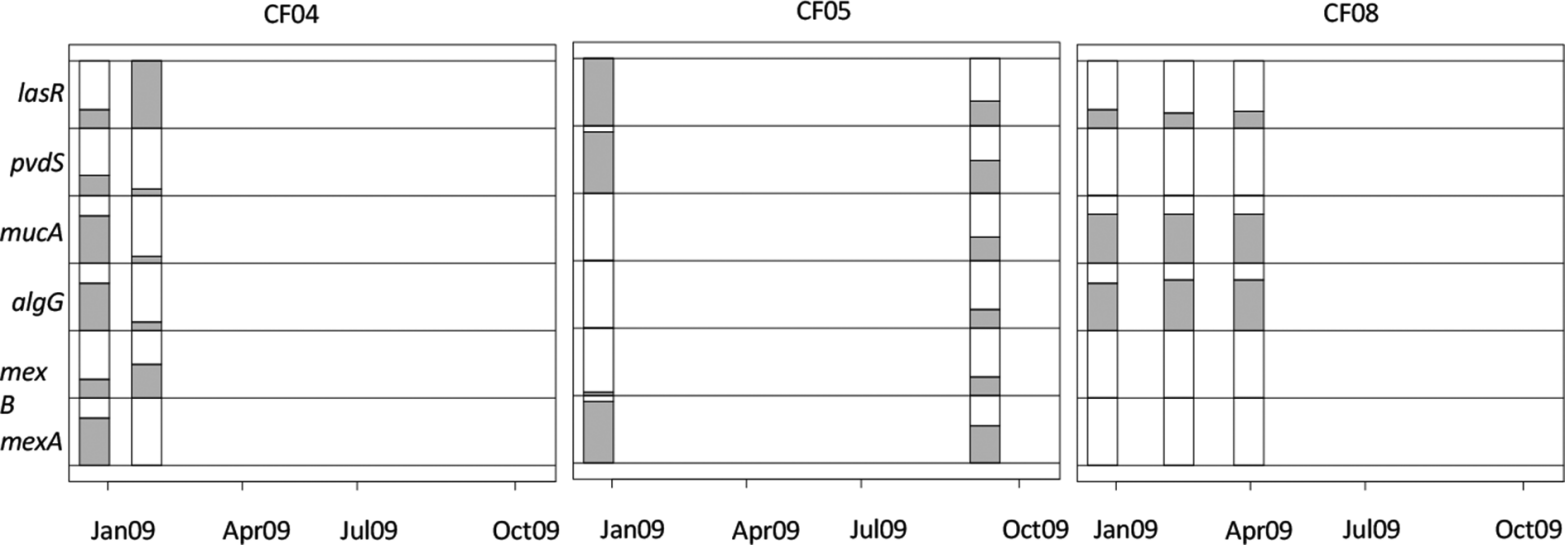
Rapid genetic changes at pathoadaptive loci. Examples of changes in abundance of mutations (shaded area) at six loci (*mexA*, *mexB*, *algG*, *mucA*, *pvdS* and *lasR*) for patients CF04, CF05 and CF08 are shown. Major changes in frequency, corresponding to changes in lineage frequency, are observed over a short time period for patient CF04, and over a longer period for patient CF05, whereas patient CF08 shows stable frequencies between the three time points.

Many loci previously proposed to underlie clinically important phenotypic traits exhibited two clear patterns. First, they were repeatedly and independently mutated in different lineages (Fig. 3). Thus, although the same genes were subject to mutations in different lineages, different nucleotide sites were mutated in each case, indicating that mutations have not arisen in one lineage and spread via recombination. Previously we have shown that rates of recombination among these samples is low [32]. Such parallel mutations highlight targets of natural selection and support the role of these loci in adaptation to the CF lung. Second, the frequency of these mutations often changed rapidly through time, concomitant with the turnover in lineages that we observed at the population level. The quorum sensing regulator *lasR*, which regulates expression of many of the *P. aeruginosa* virulence genes, was a repeated target of selection, corroborating previous observations of loss of LasR in chronic CF infections [33, 34]. Examples of the frequency dynamics in *lasR*, and other genes, are shown in Fig. 4. Patient 4 exhibited a rise in *lasR* mutation frequency from 28 to 100 % within 35 days, which was associated with the replacement of lineages detected in this patient. In patient 5, no replacement of lineages was observed but changes in the relative abundance of the two coexisting lineages was observed, concomitant with a drop in frequency of *lasR* mutations from 100 to 37 % over 8 months. By contrast, in patient 8, *lasR* mutation frequency was relatively constant in three successive samples over 3 months. Similar patterns were observed for other pathoadaptive loci: *mucA* and *algG*, associated with colony morphology variation, and linked with resistance to environmental stressors, including host defence and antibiotics [35]; *pvdS*, encoding a sigma factor required for pyoverdine synthesis to scavenge for iron during invasion, but which tends to be lost as iron becomes more available later in infection [36] or other iron acquisition systems are favoured [37]; and *mexA* and *mexB*, which encode two components of an efflux pump (MexA-MexB-OprM), associated with both antibiotic resistance and virulence [38, 39]. Loss of function mutations in *mexA* and *mexB*, as seen here and reported in other *P. aeruginosa* chronic lung infections [40], would probably lead to reduced efflux of both antibiotics and virulence factors.

## Discussion

Our data demonstrate that the genetic composition of a chronic infection by a transmissible strain changes rapidly through time, driven in large part by the turnover of genetically divergent lineages. While some lineages may be maintained within the lungs at low frequency, there is also clearly the potential for some lineages to be lost. The fact that coexistence of divergent lineages within a patient is widespread, despite loss of lineages, provides further evidence that new lineages can be acquired through the course of infection [18, 30]. The patients here were all chronically infected by the LES, attended the same clinic and were treated as a cohort [16, 17]. The LES is known to be highly transmissible (in comparison with environmental strains [9]) and these data suggest that it is readily able to super-infect susceptible individuals [30]. It is also notable that no other *P. aeruginosa* strain has been observed in these patients, which may suggest that the LES is able to outcompete other strains.

The LES is common in the UK and regions of Canada, but is only one of a number of transmissible strains that have been identified [41]. Only recently have studies started to sample multiple clones from *P. aeruginosa* infections at sufficient depth to investigate heterogeneity within infections, so it remains unclear how widespread the coexistence of multiple lineages are or their dynamics. Nevertheless, the data available indicate that other transmissible strains can harbour high levels of phenotypic heterogeneity [19]. Corresponding genomic data are available only for a small number of such strains, notably the DK02 lineage [42] but, here, stable coexistence of three independent sublineages was found in one patient [31]. Two small studies of multiple isolates from the same sputum sample suggested that coexistence of distinct lineages is not restricted to transmissible strains [43, 44]. Similarly, coexistence of distinct lineages has been reported in non-CF bronchiectasis infections [40]. It has been suggested – although not in the context of transmissible strains – that the coexistence of multiple sublineages is a consequence of the spatial heterogeneity in the lungs, whereby populations establish in physically separate populations and diverge in response to different selective pressures [45]. Evidence for the concept of regional isolation driving diversification was presented in a study of *P. aeruginosa* populations from different areas of explanted lungs from CF patients [46]. However, it has also been shown that even in a simple environment, stable coexistence can be maintained with multiple beneficial variants competing for dominance [47].

Competition between lineages may be an important feature of chronic *Pseudomonas* infections, which may drive much of the turnover of LES lineages that we observe within patients [6, 7]. It is notable that similar dynamics have recently been reported in pneumococcal infections, where negative frequency-dependent selection was shown to maintain genetic diversity [48]. With respect to patient health, many of the mechanisms used by bacteria in intra- or inter-specific competition are also involved in pathogenicity [49], suggesting traits associated with virulence may be maintained if they allow clones to defend space or resources [7, 49]. In support of this, many of the loci targeted by natural selection (evidenced by parallel mutation in multiple lineages) have previously been implicated in both clinical pathology and bacterial competition in a range of *P. aeruginosa* strains [41, 50]. In addition, selection for increased transmission may itself select for competitive ability, because following transmission LES lineages must compete with the resident lineage(s) in order to super-infect. It is known that, whereas adaptation to the lung environment has been proposed to decrease bacterial virulence in environmentally acquired strains [9], the LES and other transmissible strains are notable for maintenance of high virulence, resulting in poor patient prognosis in clinical studies [3, 14, 15]. This may therefore reflect a history of repeated transmissions selecting for the retention of virulence related traits that are important during colonisation.

Our data show that, where lineages co-occur within an infection, the majority of genetic variation is attributable to differences between lineages, rather than *in situ* diversification following infection. Super-infection by a lineage can therefore provide a substantial source of new genetic diversity, and introduce a suite of mutations at clinically relevant loci such as virulence traits or antibiotic resistance. Previously, we have shown that recombination within infections is rare and so mutations carried by a lineage remain distinct from other such lineages [32]. This linkage between loci means that a change in frequency of a mutation may be driven by selection elsewhere in the genome [20]. This could include mutations relevant to virulence increasing in frequency by hitchhiking with a linked mutation conferring antibiotic resistance. This extensive diversity present within chronic *P. aeruginosa* infections – of the LES and possibly other epidemic strains – coupled with the often rapid turnover in lineages, suggests that such genetic dynamics may make the clinical outcome of interventions unpredictable.

Longitudinal population genomic sequencing of a transmissible *P. aeruginosa* strain from multiple patients has revealed the coexistence of highly diverged lineages within individual patients and rapid turnover of lineages over time. The acquisition of new lineages by transmission represents a major influx of genetic diversity to a chronic infection and greater than that observed for diversification *in situ*. The population genomic structure of infections showed rapid shifts as lineages competed with or displaced each other. Because each lineage consists of a set of linked loci affecting a suite of phenotypic traits including key pathoadaptive loci, transmission and lineage turnover dynamics lead to much faster change than might be expected by mutation and selection during adaptation of an environmental strain to the CF airway. Our results emphasize the importance of infection control among cohorts of CF patients to prevent the acquisition of novel *P. aeruginosa* lineages and the subsequent competition between lineages, which may exacerbate the pathology associated with transmissible strains [8, 14].

## Data bibliography

1. Williams, D European Nucleotide Archive PRJEB6642 (2013).
2. Paterson, S European Nucleotide Archive PRJEB20763 (2017).

## Supplementary Data

214 Supplementary File 1
