# Supplementary material for: Transmission and lineage displacement drive rapid population genomic flux in cystic fibrosis airway infections of a Pseudomonas aeruginosa epidemic strain
Source: Microb Genom. 2018 Mar 16;4(3):e000167. doi: 10.1099/mgen.0.000167 (PMC5885019; doi:10.1099/mgen.0.000167)
Supplement: Supplementary File 1 [file mgen-4-167-s001.pdf]

Supplementary material

Transmission and lineage displacement drive rapid population genomic flux in cystic fibrosis airway infections of a *Pseudomonas aeruginosa* epidemic strain

Supplementary Methods

Inference of divergent lineages in single isolates and in pooled data

Figure S1 shows a simplified phylogeny inferred from single isolates from patient CF03 taken in the first January 2009 sample. SNPs can be classified as (a) common to all isolates within a sample, but having arisen since the most recent common ancestor (MCRA) of the set of isolates from all patients in the study, (b) and (c) arising on the two branches (lineages) leading from the common ancestor of samples within that sample, and that are common to all isolates within that branch. Finally, SNPs may be unique to a lineage but not carried by all isolates within a lineage, shown by the grey triangles. In the example shown, the branch lengths of b and c and are longer than that of a, indicating the presence of divergent lineages within a single patient.

Figure S2 shows the distribution of SNP frequencies estimated from pooled sequencing data among 5 samples from patient CF03 taken over the course of 2 months. SNPs corresponding to branches b and c form distinct peaks from which the proportion of clones from each lineage can be estimated. SNPs having arisen by subsequent diversification (i.e. the grey triangles in Figure S1) appear at a lower frequencies, since they are carried by only a few isolates. Further details and examples can be found in (1).

It is also worth noting that the reference sequence chosen to map reads against does not affect the phylogenies, since these rely on phylogenetically informative SNPs (ie those that are not fixed across the sample). For example using LES431 as a reference instead of LESB58, the 22 genes shown in Figure 3 only exhibit 2 SNP differences between the two

strains and these are fixed differences that do not contribute to inference of topology or branch length within the isolates sequenced here. Systematic differences dues to mobile elements are specifically excluded in our methods.

References

1. Williams D, Evans B, Haldenby S, Walshaw MJ, Brockhurst MA, Winstanley C, et al. Divergent, coexisting *Pseudomonas aeruginosa* lineages in chronic cystic fibrosis lung infections. Am J Respir Crit Care Med. 2015;191:775–85.

2. Fothergill JL, Ledson MJ, Walshaw MJ, McNamara PS, Southern KW, Winstanley C. Comparison of real time diagnostic chemistries to detect *Pseudomonas aeruginosa* in respiratory samples from cystic fibrosis patients. J Cyst Fibros. 2013;12:675–81.

Table S1. Summary of patients and samples used in study. Values for forced expiratory volume in one second (FEV1) and body mass index (BMI) for each patient at the time of the first sample (January 2009) are shown.

Patient	Sex	Age (yr)	FEV1 (%)	BMI
CF1	F	30	79	21
CF3	F	20	36	28
CF4	M	31	43	20.5
CF5	M	19	84	23
CF7	F	24	37	17
CF8	F	25	37	18
CF9	F	28	30	24

Table S2 Summary of samples and isolates sequenced in this study. Samples from which isolates were taken for genome sequencing are shown, with the date of sample and the number of isolates from each sample that were individually sequenced, and the number of isolates sequenced within each pool. To validate methods, sample CF03-S02 was sequenced for each of 40 isolates indivually and as a pool (1).

Patient	Sample date	Label	Number of single isolates	Number of isolates in pool
CF01	06/01/2009	S01	2	40
CF01	22/09/2009	S04	2	40
CF03	06/01/2009	S01	2	40
CF03	20/01/2009	S02	40	40
CF03	25/01/2009	A01	2	40
CF03	27/01/2009	A02	2	40
CF03	03/02/2009	A03	2	40
CF04	06/01/2009	S01	2	40
CF04	10/02/2009	S02	2	40
CF05	06/01/2009	S01	2	40
CF05	22/09/2009	S04	2	40
CF07	06/01/2009	S01	2	40
CF07	17/02/2009	S04	2	40
CF07	23/02/2009	A01	2	40
CF07	10/03/2009	A03	2	40
CF08	06/01/2009	S01	2	40
CF08	26/02/2009	A02	2	40
CF08	14/04/2009	S03	2	40
CF09	06/01/2009	S01	2	40
CF09	01/12/2009	S10	2	40
CF09	28/01/2010	A02	2	40

Table S3 Primers used in qPCR assays

Primer	Sequence (5′-3′)	Target	Amplicon (bp)	Limit of Detection	Efficiency	R^2	Reference
gyrPA F1	CCTGACCATCCGTCGCCACAA	*gyrB* (*P. aeruginosa*)	221	$4.7x10^1$	1.02	0.99	Ref 2
gyrPA R1	CGCAGCAGGATGCCGACGC						
L5_F	CGGATCAAGCAGAGGCCG	Lineage D	209	$2.4x10^3$	1.01	0.99	This Study
L5_R	GTGCCGGACGATCAGGTC						
L11_F	GTAACTCCGACGGCAGACC	Lineage I	207	$1.0x10^4$	0.90	0.99	This Study
L11_R	TCCTTGGTCATGCTGCCG						

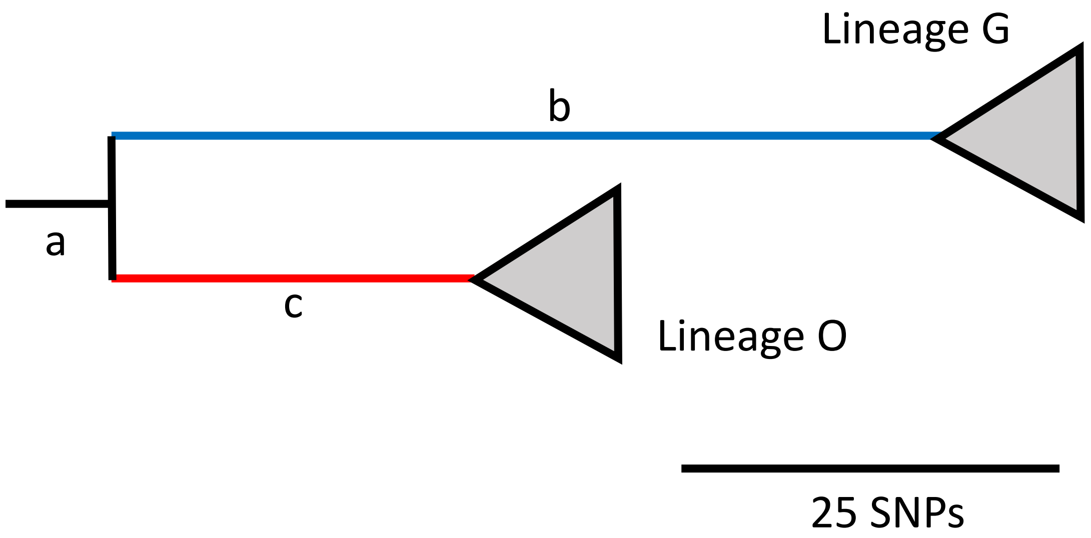

Figure S1. Illustration of a phylogeny of divergent lineages within a sample. Data for the first sample from patient CF03 are shown. (a) is the branch length from the most recent common ancestor (MRCA) of the epidemic to the MRCA of the isolates within the sample. (b) and (c) are branch lengths of the first bifurcation after the MRCA of the isolates within the sample.

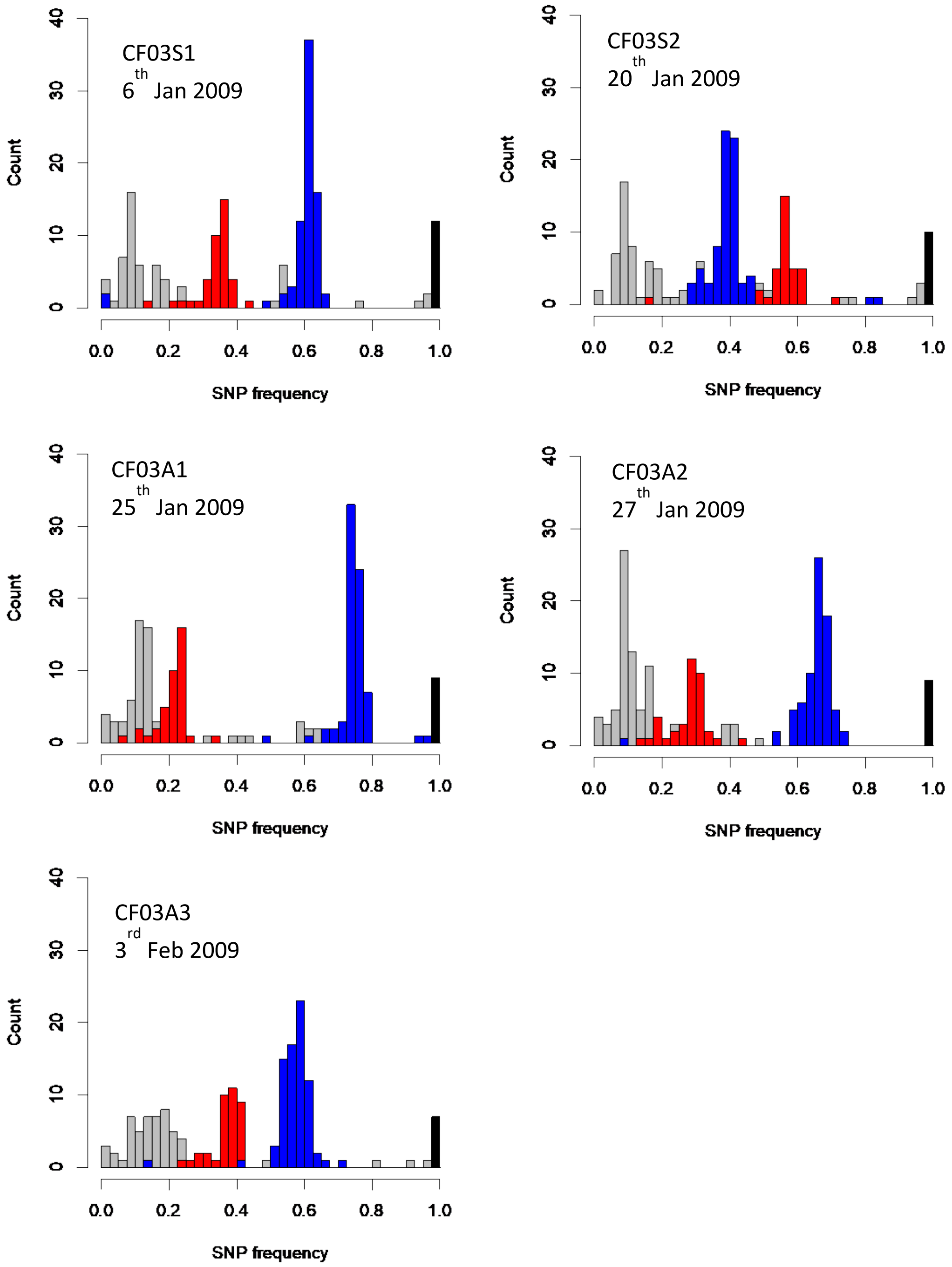

Figure S2 Frequency distribution of SNPs correponding to major lineages within samples. The figure shows data for 5 samples taken from patient CF03 and represents the number of SNPs (y-axis) occurring at different frequencies (x-axis). SNPs unique to lineages G and O are shown in blue and red, respectively. SNPs common to both lineages (branch (a) in Figure S1) are shown in black. SNPs that are polymorphic within lineages (grey triangles in Figure S1) are shown in grey.

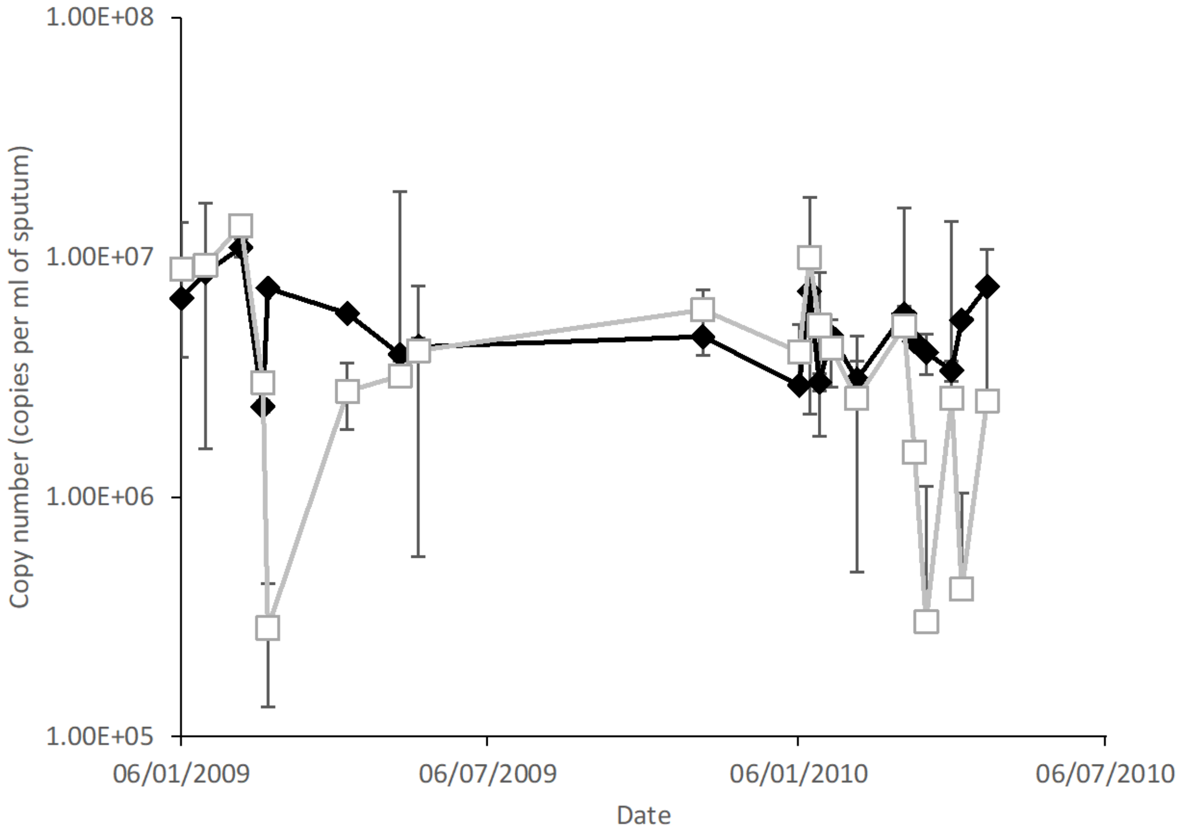

Figure S3. Fluctuations in lineage D detected by qPCR in CF patient CF7 sputum. Mean and standard deviation are plotted. Black diamonds are total *Pseudomonas*; grey triangle are lineage D.

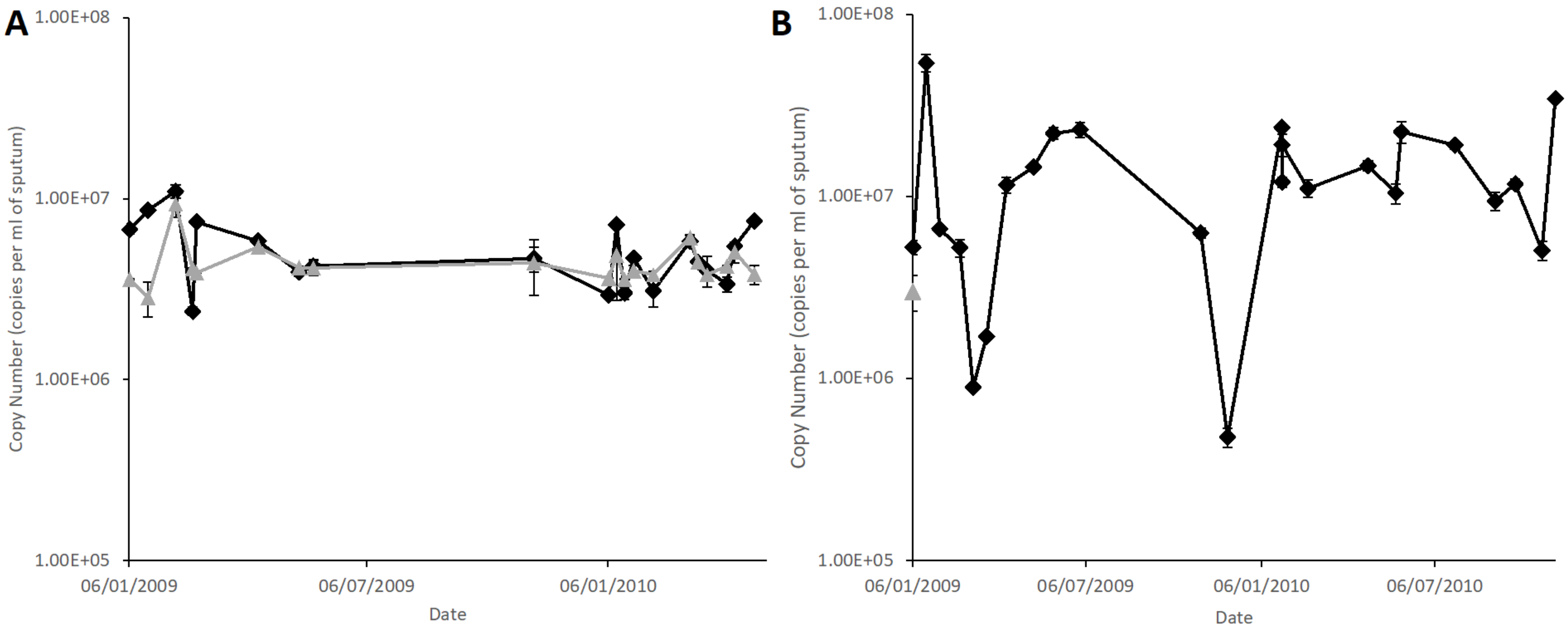

Figure S4. Fluctuations in lineage I detected by qPCR in CF patient sputum. Mean and standard deviation are plotted for A) Patient CF7 and B) Patient CF9. Black diamonds are total *Pseudomonas*; grey triangle are lineage I.
